# Leptin Induces an Inflammatory Phenotype in Lean Wistar Rats

**DOI:** 10.1155/2009/738620

**Published:** 2010-01-26

**Authors:** Monique Allman, Mathew Wallace, Latausha Gaskin, Chantal A. Rivera

**Affiliations:** Department of Molecular and Cellular Physiology, Louisiana State University Health Sciences Center, 1501 Kings Hwy, Shreveport, LA 71130, USA

## Abstract

The present study addressed the hypothesis that leptin promotes leukocyte trafficking into adipose tissue. Accordingly, male Wistar rats were treated with saline or recombinant rat leptin (1 mg/kg) via the tail vein. Leukocyte trafficking in mesenteric venules was quantified by intravital microscopy. Treatment with leptin resulted in a 3- and 5-fold increases in rolling and firm adhesion, respectively. Compared to vehicle controls, leptin enhanced mRNA levels of IL-6 (8-fold) and MCP-1 (5-fold) in mesenteric adipose tissue (MAT). Similar increases in these markers were observed in mesenteric venules and in liver. Finally, the direct effect of leptin was assessed in C3A hepatocytes treated with leptin for 24 hours (7.8 ng/mL–125 ng/mL). Consistent with observations in vivo, production of ICAM-1, MCP-1, and IL-6 by hepatocytes was increased significantly. These findings support the hypothesis that leptin directly initiates inflammation in the local environment of mesenteric adipose tissue as well as systemically.

## 1. Introduction

Obesity is reaching epidemic proportions in the United States as well as many other countries. The degree of adiposity, visceral adiposity in particular, correlates with the extent of obesity-associated comorbid conditions such as hypertension, diabetes, and cardiovascular disease. Moreover, it is believed that abdominal adiposity plays a key role in the chronic inflammatory state that predisposes to these comorbidities [1–3]. For example, a positive correlation exists between adiposity (i.e., BMI and % body fat), IL-6 protein levels in serum, as well as mRNA levels of tumor necrosis factor-alpha (TNF-*α*) and interleukin-6 (IL-6) in abdominal adipose tissue [4, 5]. Consequently, recent experimental and clinical research has focused on elucidating the contribution of adipose tissue to the development of systemic pathologies. 

Growing adipose tissue depots are characterized by enhanced macrophage content and the induction/activation of several proinflammatory genes. Adipocyte conditioned medium induces the expression of leukocyte-endothelial cell adhesion molecules, and stimulates monocyte diapedesis across monolayers of cultured endothelial cells [6]. However, specific factors responsible for vascular inflammation observed during the growth of adipose cells have not been fully elucidated. Leptin is a hormone produced in adipocytes that regulates both food intake and energy expenditure. Importantly, it appears that serum leptin may represent an independent predictor of the severity of illnesses associated with obesity. In fact, hyperleptinemia is an independent predictor of nonalcoholic steatohepatitis (NASH) in obese humans, and the severity of steatosis, hepatocyte ballooning, as well as nonalcoholic fatty liver disease (NAFLD) activity scores correlate with serum leptin levels [7]. Based on findings linking leptin to systemic inflammation, it is hypothesized that leptin can directly modulate the inflammatory response in the vasculature and liver. Accordingly, we performed intravital microscopic analysis to examine the direct effects of bolus (1 mg/kg) intravenous leptin administration on leukocyte-endothelial cell interactions in the rat mesenteric microvasculature. This dose was chosen in an attempt to mimic hyperleptinemia as described by others [8, 9]. The effects of hyperleptinemia on the liver in vivo and on cultured hepatocytes were also monitored. Our results provide the first real-time evidence that leptin promotes leukocyte trafficking in the microvasculature and directly stimulates a proinflammatory phenotype in hepatocyte cultures.

## 2. Materials and Method

### 2.1. Materials

Human C3A hepatocytes and culture reagents were purchased from ATCC, (Manassas, VA). Leptin was obtained from Sigma-Aldrich (St. Louis, MO). An RNeasy Mini Kit, for RNA extraction, was purchased from Qiagen (Valencia, CA). DuoSet ELISA kits for Human ICAM-1, IL-6, and MCP-1 as well as ELISA kits for rat Adiponectin were purchased from R&D Systems (Minneapolis, MN). Gene expression Assays for real-time PCR were obtained from Applied Biosystems (Foster City, CA). 

### 2.2. Animal Treatment and Intravital Microscopy

Male Wistar rats were administered saline (controls; *n* = 6) or recombinant rat leptin (1 mg/kg; *n* = 6) via the tail vein. This dose was based on previously reported doses for rodent studies [8, 9]. After 4 hours or 24 hours, intravital microscopy was used to monitor and quantify leukocyte trafficking as described previously [10]. Briefly, while under ketamine hydrochloride (150 mg/kg i.m.) and xylazine (7.5 mg/kg i.m.) anesthesia, a laparatomy was performed and a small portion of the mesentery was exteriorized. An Olympus IX71 inverted microscope equipped with a Sony DSP 3CCD color video camera was used to record each intravital experiment for offline analysis. Vessels chosen for analysis were approximately 20–50 *μ*m in diameter. At least 3 non-overlapping regions were analyzed for each rat. Rolling leukocytes were defined as cells moving at a velocity significantly slower than center line velocity. Adherent leukocytes were determined as cells that were completely stationary for at least 30 seconds.

### 2.3. Hepatic Histology

At the end of the intravital analysis, samples of liver were flash-frozen in liquid nitrogen and stored at −80°C for RNA extraction. Additional samples were preserved in zinc fixative, processed with paraffin, and 4 *μ*m sections were stained with hematoxylin and eosin. Hepatic injury and inflammation were assessed by one of the authors blinded to the study design.

### 2.4. Cell Culture

Human C3A hepatocytes were grown to confluence in Eagle's Minimum Essential Media (EMEM) supplemented with 10% Fetal Bovine Serum (FBS). When cultures reached confluence, C3A cells were incubated overnight in EMEM medium without FBS for 24 hours prior to treatment with recombinant human leptin (7.8–125 ng/mL). After an additional 24 hours period in the presence of leptin, the culture medium was collected and the cells were harvested. The cell pellet was stored at −80°C until used for RNA extraction. 

### 2.5. Measurement of Inflammation Markers

At the time of sacrifice, samples of whole blood were collected from the vena cava of saline controls and leptin-treated rats. Serum levels of the anti-inflammatory adipokine adiponectin were determined by ELISA. Soluble ICAM-1, IL-6, and MCP-1 were measured in samples of culture medium collected 24 hours after leptin exposure using Duoset ELISA kits according to the manufacturer's instructions.

### 2.6. RNA Isolation and Reverse Transcription

Total RNA was extracted from frozen samples of mesenteric adipose tissue, mesenteric connective tissue containing microvessels, and liver using the RNeasy Mini kit from Qiagen. The integrity and concentration of each total RNA sample were determined by spectrophotometry. A 500 ng aliquot of total RNA was reverse-transcribed to cDNA in 25 *μ*L of reaction mixture containing 25 mM MgCl_2,_ 10 mM dNTP mix, 50 *μ*M Random hexamers, 20 U/uL RNase inhibitor, and 50 U/uL multiscribe reverse transcriptase. Reaction mixtures were incubated in a Mastercycler-personal (Eppendorf, Westbury, NY) for 10 minutes at room temperature followed by 60 minutes at 42°C and 5 minutes at 95°C.

### 2.7. Quantitative Real-Time Polymerase Chain Reaction

Relative mRNA expression of ICAM-1, MCP-1, IL-6, and P-selectin was determined using predeveloped assays for real-time PCR (Applied Biosystems, Foster City, CA). Analysis of each target mRNA was performed in duplicate in separate wells and normalized to amplification of 18S ribosomal RNA subunit. To confirm the absence of genomic DNA contamination, samples without the reverse transcription were also analyzed. The reactions were performed using an ABI Prism 7700 Sequence Detection System with the following cycle parameters: 50°C for 2 minutes, 95°C for 10 minutes followed by 40 cycles of 95°C for 15 seconds and 60°C for 1 minute. Raw data were analyzed using the ABI Prism Sequence Detection 1.9.1 software. The relative amount of mRNA was calculated using the comparative threshold cycle (C_t_) method as described previously [11]. 

### 2.8. Statistical Analysis

Data are presented as mean ± SEM of 4–6 observations per treatment group. Statistical analysis was performed using student's *t*-test or one-way ANOVA with Tukey's multiple comparisons test where appropriate as indicated in the figure legends; *P *< .05 was selected as the level of significance.

## 3. Results

### 3.1. The Effects of Leptin on Leukocyte Trafficking In Vivo

**Figure 1:**
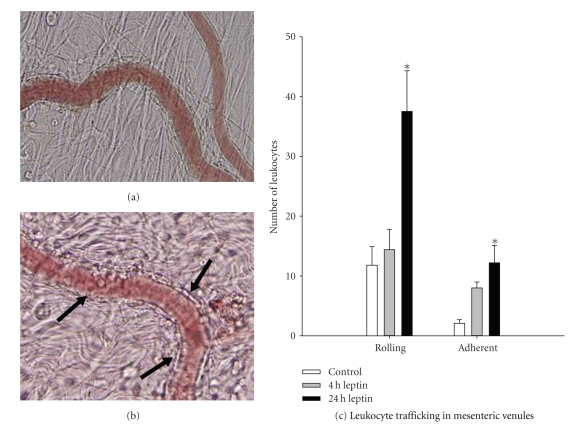
*Leukocyte trafficking in the mesenteric microvasculature*. Wistar rats (200–250 g) were subjected to intravital microscopic analysis of leukocyte trafficking in mesenteric postcapillary venules. (a) Occasional rolling and adherent leukocytes were observed in mesenteric venules in saline controls. (b) Mesenteric venules 24 hours after leptin administration (1 mg/kg i.v.) displayed a marked enhancement in leukocyte trafficking as denoted by arrows. (c) Summary of control and leptin-stimulated leukocyte trafficking. Data are mean ± SEM analyzed by one-way ANOVA; *P < .05 compared to control.

The in vivo response to leptin was investigated using video microscopy of leukocyte rolling and firm adhesion in the mesenteric microvasculature 24 hours after leptin treatment. Representative photomicrographs of the vasculature are presented in Figures 1(a) and 1(b). As summarized in Figure 1(c), there was a significant 3-fold increase in rolling as well as adherent leukocytes along the vascular endothelium compared to saline-treated control rats 24 hours after leptin treatment. 

**Figure 2:**
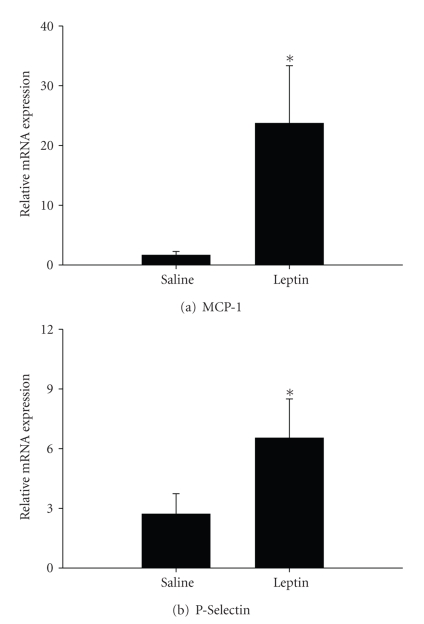
*The relative mRNA expression of MCP-1 and P-selectin*. Expressions of (a) MCP-1 and (b) P-selectin were assessed using pre-developed assays for real-time PCR according to the manufacturer's instructions (Applied Biosystems). Values were calculated using a comparative C_t_ method and presented as mean ± SEM of at least 4 observations/group. Statistical analysis was performed using student's *t*-test. *P < .05 saline controls.

To further index the vascular response to leptin, mesenteric tissue containing microvessels was isolated separately from gut and adipose tissue for analysis of mRNA levels of the inflammatory mediators MCP-1 and P-selectin. Expression of both markers was enhanced significantly by more than 25-fold and 2-fold, respectively, in rats administered leptin (Figure 2).

### 3.2. Effect of Leptin on Adipose Tissue

**Figure 3:**
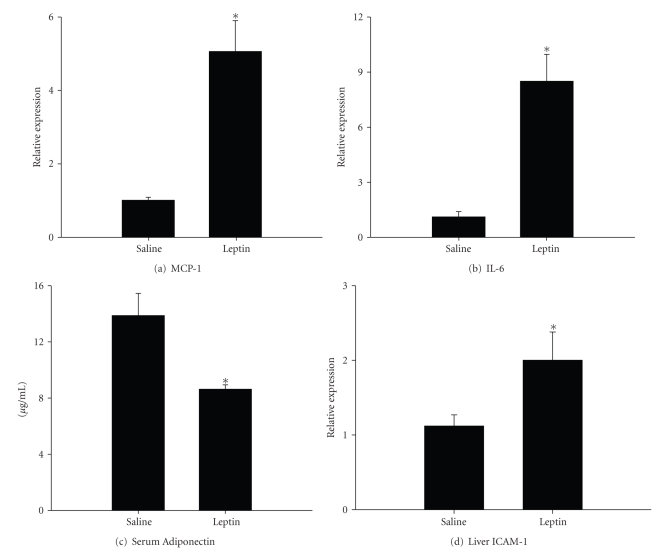
*Effects of leptin on adipose tissue, serum adiponectin, and liver*. Samples of mesenteric adipose tissue, serum and liver were collected from leptin-treated rats (1 mg/kg via the tail vein) and saline controls, and the following parameters were measured: Expressions of (a) MCP-1 and (b) IL-6 in samples of mesenteric adipose tissue were assessed using predeveloped assays for real-time PCR according to the manufacturer's instructions (Applied Biosystems). Values were calculated using a comparative C_t_ method. (c) Serum levels of adiponectin were measured by ELISA. (d) Hepatic mRNA expression of ICAM-1 was measured by real-time PCR. Data are presented as mean ± SEM of at least 4 observations/group. Statistics were performed using Student's *t*-test *P < .05 compared to saline controls.

To determine whether leptin plays a role in adipose tissue inflammation, expression of MCP-1 and IL-6 was assessed in samples of mesenteric adipose tissue collected 24 hours after intravenous leptin treatment. Compared to control rats administered saline, mRNA levels of MCP-1 were increased nearly 6-fold (Figure 3(a)). Expression of IL-6 was also significantly enhanced by approximately 9-fold above control levels (Figure 3(b)). Serum samples were analyzed to determine the presence of adiponectin. As shown in Figure 3(c), the amount of adiponectin protein in serum of control rats was approximately 13.9 ± 1.6 mg/mL, but was decreased significantly to 8.6 ± 0.3 mg/mL by leptin. Thus, leptin stimulates expression of proinflammatory mediators while inhibiting the production of the potentially beneficial hormone adiponectin.

### 3.3. Effects of Leptin on the Liver

Since obesity and hyperleptinemia are also associated with the hepatic disease known as nonalcoholic steatohepatitis, we next determined if leptin administration promotes hepatic inflammation. Histological samples of liver collected at 4 hours or 24 hours after leptin injection did not display an overt accumulation of leukocytes or injury (data not shown). On the other hand, PCR analysis of ICAM-1 expression revealed a 2-fold increase in mRNA levels at the 24 hours time point (Figure 3(d)), suggesting that leptin promotes hepatic inflammation and the potential for leukocyte accumulation.

### 3.4. Effects of Leptin on Cultured Hepatocytes

**Figure 4:**
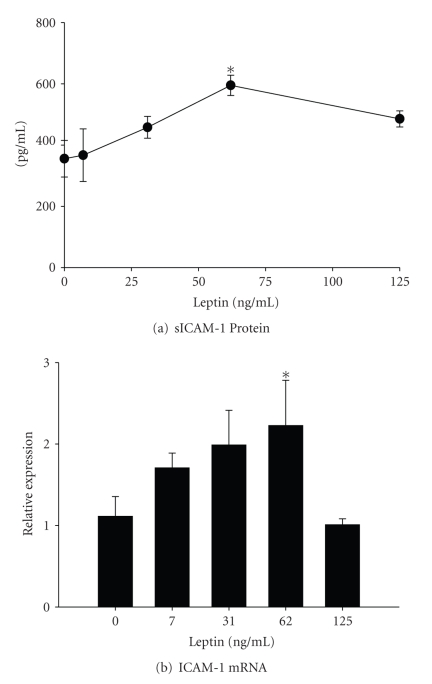
*Effect of leptin on hepatocytes*. Human C3A hepatocytes were cultured in the presence of leptin (0–125 ng/mL) for 24 hours. (a) The release of soluble ICAM-1 into the culture medium and (b) ICAM-1 mRNA levels was quantified by ELISA and real-time PCR, respectively. Statistics were performed using one-way ANOVA *P< .05 compared to vehicle control cultures.

**Figure 5:**
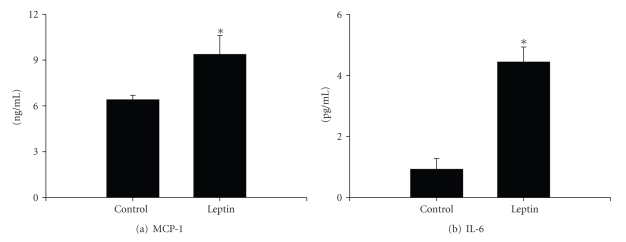
*Effect of leptin on hepatocyte cytokine and chemokine release*. Protein levels of IL-6 and MCP-1 produced by hepatocytes cultured in the presence of 62.5 ng/mL leptin for 24 hours were quantified using a high-sensitivity Quantikine ELISA kit (R&D systems, Inc). Data are presented as mean ± SEM of at least 4 observations/group. Statistical analysis was performed using Student's *t*-test *P< .05 compared to vehicle control cultures.

The C3A human hepatocyte cell line was used to determine if leptin exposure could directly stimulate a proinflammatory phenotype. After up to 24 hours in culture, protein and mRNA expression of ICAM-1 was monitored using ELISA and real-time PCR, respectively. Protein levels of sICAM released by C3A cells increased in a dose-dependent manner (Figure 4(a)). Leptin maximally increased sICAM release by approximately 1.5-fold at a dose of 62.5 ng/mL leptin. The mRNA levels of ICAM-1 were also increased significantly compared to vehicle control cultures (Figure 4(b)). Because the peak effect of leptin was observed at a concentration of 62 ng/mL, the remaining experiments were performed using only this dose. Finally, protein levels of MCP-1 and IL-6 were quantified using ELISA to further index the inflammatory state in hepatocytes. In the samples of culture medium collected from untreated cells, the amount of MCP-1 produced was approximately 6.4 ± 0.3 ng/mL (Figure 5(a)). Leptin treatment increased MCP-1 concentrations to 9.4 ± 1.2 ng/mL. In addition, the production of IL-6 was significantly enhanced by approximately 5-fold (4.4 ± 0.5 pg/mL) compared to vehicle control cultures (09. ± 0.3 pg/mL; Figure 5(b)).

## 4. Discussion

Leptin is 16 kDa protein primarily known for regulation of caloric load via appetite suppression and energy expenditure under normal physiological conditions. Mutations in the leptin (*O* *b*) or leptin receptor (*O* *b* *r*) genes inhibit the appropriate production of or response to leptin, respectively. Although rare in humans, these mutations render affected individuals obese. On the other hand, obesity as a result of poor life style choices (e.g., hyperphagia, sedentary behavior) is commonly associated with hyperleptinemia. Several studies have shown that serum leptin levels correlate with generalized obesity indexed by BMI and even more specifically with abdominal adiposity. Considine et al. (1996) were among the first to report a positive relationship between leptin and adiposity [12]. They reported significantly higher serum leptin levels of 31.3 ± 24.1 ng/mL in obese subjects compared to lean controls (7.5 ± 9.3 ng/mL). Protein levels as well as leptin gene expression in adipocytes strongly correlated with % of body fat, and hyperleptinemia was improved with weight loss. Receptors for leptin are expressed throughout the body and leptin binding to these receptors has been shown to stimulate adaptive as well as innate immune response as reviewed elsewhere [9, 13, 14]. For example, leptin has been shown to participate in the pathogenesis of autoimmune encephalomyelitis causing a profound down-regulation of the content of regulatory T lymphocytes in the CNS fluid [15, 16]. With respect to innate immunity, leptin promotes monocyte/macrophage activation states, a process that likely triggers neutrophil chemotaxis [14, 17]. Moreover, this adipokine elicits the activation of NFkB [18], a transcription factor that initiates the synthesis of cell adhesion glycoprotein molecules (e.g., ICAM-1) and chemokines (e.g., MCP-1). In the setting of diet-induced obesity, serum leptin levels correlate directly with the presence of comorbidities such as cardiovascular disease and liver disease. These findings have warranted investigations into the possible pathogenic roles of leptin in the genesis of comorbidities associated with obesity. Indeed, the low-grade chronic inflammatory state that accompanies obesity is thought to be a mitigating factor in the development of cardiovascular abnormalities, liver disease, and various other pathological conditions. 

In terms of cardiovascular disease, plasma leptin levels are reported to be an independent predictor of early atherosclerotic events such as intima-media thickness [19, 20]. Previous studies in rodent models provide insight into the influences of leptin on vascular disease; however, results are equivocal and underlying mechanisms of vascular reactivity remain to be elucidated as reviewed elsewhere [21]. For example, Valeric et al. (2009) found that leptin deficient ob/ob mice were more susceptible to stroke induced by permanent cerebral artery occlusion [18]. In contrast, Terao et al. reported exacerbated vascular injury that was associated with enhanced leukocyte and platelet trafficking in microvessels [22]. The acute (30 minutes) occlusive event used in the latter study is likely more relevant to clinical stroke. Moreover, leptin reconstitution in ob/ob mice further augmented infarct volume, providing evidence that leptin has an adverse influence on the vasculature. In the present study, we administered a bolus dose of leptin in an attempt to mimic the hyperleptinemic state that accompanies obesity. Our findings provide strong support of a role for hyperleptinemia in the development of vascular and tissue injury, however we focused specifically on inflammatory changes in the microcirculation and not vascular reactivity. Our data indicate that bolus intravenous leptin administration can directly stimulate leukocyte and platelet interactions with the endothelial lining of the mesenteric microvasculature, most likely via alterations in proinflammatory cytokines and chemokines. To our knowledge, this is the first real-time in vivo demonstration of the direct inflammatory effects of hyperleptinemia in lean rats in the absence of any underlying pathological conditions. 

Nonalcoholic steatohepatitis is a cryptogenic form of liver disease that is often observed in obese patients without identifiable causes such as alcohol abuse, drug toxicity, or viral infection. As the name suggests, NASH is histologically indistinguishable from alcoholic hepatitis and is characterized by fat accumulation in hepatocytes, mixed cell type inflammation, focal necrosis, and fibrosis. This disease has been observed worldwide and the prevalence of NASH is estimated to be as high as 40–100% in obese adults as reviewed elsewhere [23]. Adiposity is clearly one of the most studied risk factors for NASH [24, 25]. It has been proposed that abdominal adiposity is particularly related to disease state [1–3]. With respect to on advanced disease, a significant positive correlation has been identified between advanced fibrosis stage and indices of adiposity (e.g., BMI, blood cholesterol and LDL levels) [24–26]. The precise mechanistic basis of NASH is not known; however previous findings suggest that leptin may be an important link between adiposity and liver injury in obese people. Although it is primarily secreted by adipocytes, recent reports demonstrated that leptin was also expressed by activated stellate cells [9]. Interestingly, leptin enhanced the expression of cytokines related to the induction of fibrosis in the liver (e.g., transforming growth factor-*β*, TGF-*β*) and induced the overproduction of matrix proteins that are components of fibrosis scaring such as collagen *α*I-1 [9, 27–29]. Moreover, in animal models of NASH, spontaneous conversion from simple fat accumulation in hepatocytes to injury and fibrosis does not occur in leptin-deficient ob/ob mice [30]. Herein, we report that exposure of C3A human hepatocytes to leptin induced a proinflammatory phenotype characterized by enhanced expression of cytokines (IL-6), chemokines (MCP-1), and the adhesion molecule ICAM-1. Leptin administration in vivo significantly enhanced hepatic ICAM-1 expression. Taken together, the present findings provide compelling evidence of a direct proinflammatory effect of hyperleptinemia on the liver. In addition, we found that bolus leptin administration resulted in a decrease in circulating serum adiponectin levels, an adipocyte-derived hormone widely believed to have anti-inflammatory properties. In contrast to leptin, recent studies have identified an inverse relationship between NASH severity and serum adiponectin levels [31–33]. Studies in animal models of NASH have confirmed that hypoadiponectinemia augments steatohepatitis, while transgenic mice that overexpress this hormone were protected from pathology [34, 35]. Although a correlation of the serum adiponectin/leptin ratio with disease pathogenesis has been noted in humans with NASH [31, 33], the exact mechanistic link between these two adipokines remains to be elucidated. 

## 5. Conclusion

**Figure 6:**
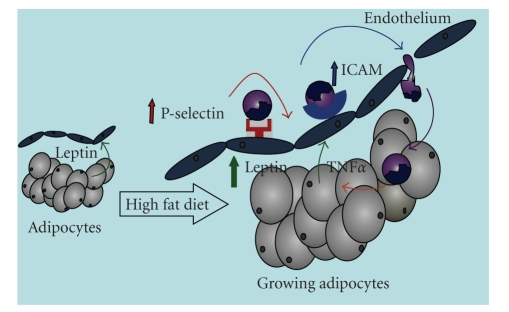
*Working hypothesis*. Although leptin is constitutively expressed and released by adipocytes, its expression is enhanced during weight gain/fat deposition. We hypothesize that augmented leptin release from growing adipocytes up-regulates adhesion molecules on endothelial cells, which promote the subsequent leukocyte invasion into adipose tissue. The production of inflammatory cytokines by invading leukocytes potentiates leptin synthesis, which will ultimately have detrimental effects on the local microvasculaure. Since blood leaving the mesentery drains directly into the portal circulation, leptin likely plays an important role in the development of obesity-associated nonalcoholic steatohepatitis.

Taken together with previous reports in rodent models as well as humans, data presented herein demonstrate the potential role for leptin in the inflammatory process associated with obesity. Our working hypothesis is outlined schematically in Figure 6. Briefly, in an obesigenic environment, hyperleptinemia results from growing adipose tissue depots. The overproduction of leptin stimulates the synthesis of inflammatory mediators such as MCP-1 and ICAM-1, which promote leukocyte trafficking in the local microvasculature. As obesity, adipose tissue inflammation, and the hyperleptinemic state become more exaggerated, systemic inflammation ensues. Thus, although leptin is thought to have some neuroprotective effects [18, 36] and has been the focus of several hundred pharmacological intervention studies for weight loss as reviewed elsewhere [37], therapeutic efficacy of this adipokine will likely be proven to be very limited in the setting of obesity due to adverse effects on immune function.
